# The Effect of Diabetes-Associated Autoantigens on Cell Processes in Human PBMCs and Their Relevance to Autoimmune Diabetes Development

**DOI:** 10.1155/2013/589451

**Published:** 2013-06-12

**Authors:** Jana Vcelakova, Radek Blatny, Zbynek Halbhuber, Michal Kolar, Ales Neuwirth, Lenka Petruzelkova, Tereza Ulmannova, Stanislava Kolouskova, Zdenek Sumnik, Pavlina Pithova, Maria Krivjanska, Dominik Filipp, Katerina Stechova

**Affiliations:** ^1^Department of Paediatrics, 2nd Faculty of Medicine, Charles University in Prague and University Hospital Motol, V Uvalu 84, 15006 Prague, Czech Republic; ^2^Central European Biosystems, Nad Safinou II 365, 25242 Vestec, Czech Republic; ^3^Laboratory of Genomics and Bioinformatics, Institute of Molecular Genetics AS CR, Prague, Czech Republic; ^4^Department of Immunobiology, Institute of Molecular Genetics, Czech Academy of Science, Videnska 1083, 14220 Prague, Czech Republic; ^5^Department of Internal Medicine, 2nd Faculty of Medicine, Charles University in Prague and University Hospital Motol, V Uvalu 84, 15006 Prague, Czech Republic

## Abstract

Type 1 Diabetes (T1D) is considered to be a T-helper- (Th-) 1 autoimmune disease; however, T1D pathogenesis likely involves many factors, and sufficient tools for autoreactive T cell detection for the study of this disease are currently lacking. In this study, using gene expression microarrays, we analysed the effect of diabetes-associated autoantigens on peripheral blood mononuclear cells (PBMCs) with the purpose of identifying (pre)diabetes-associated cell processes. Twelve patients with recent onset T1D, 18 first-degree relatives of the TD1 patients (DRL; 9/18 autoantibody positive), and 13 healthy controls (DV) were tested. PBMCs from these individuals were stimulated with a cocktail of diabetes-associated autoantigens (proinsulin, IA-2, and GAD65-derived peptides). After 72 hours, gene expression was evaluated by high-density gene microarray. The greatest number of functional differences was observed between relatives and controls (69 pathways), from which 15% of the pathways belonged to “immune response-related” processes. In the T1D versus controls comparison, more pathways (24%) were classified as “immune response-related.” Important pathways that were identified using data from the T1D versus controls comparison were pathways involving antigen presentation by MHCII, the activation of Th17 and Th22 responses, and cytoskeleton rearrangement-related processes. Genes involved in Th17 and TGF-beta cascades may represent novel, promising (pre)diabetes biomarkers.

## 1. Introduction

Type 1 Diabetes (T1D) is considered to be a T-helper- (Th-) 1 autoimmune disease and is characterised by a lack of insulin, which is caused by the autoimmune destruction of insulin-producing pancreatic beta cells [1, 2]. Th1 lymphocytes are responsible for the infiltration of the islets of Langerhans and for the cytokine release that facilitates the destruction of beta cells by cytotoxic (Tc) lymphocytes. Due to this progressive damage, there is either insufficient or no production of insulin, leading to the first clinical signs of T1D. At the first appearance of clinical symptoms, most notably those associated with hyperglycaemia, nearly 80% of the beta cells have been destroyed, rendering the individual dependent on insulin injections [2, 3].

In patients presenting with recent T1D onset, there are various interventions that may stop, or at least delay, pancreatic beta cell destruction; however, these therapies are unable to reverse the patient's lifelong dependency on insulin injections because beta cell proliferation and their capacity for regeneration are limited. To save sufficient beta cell masses, these therapies should be used in the clinically silent prediabetes phase; however, it is difficult to identify suitable candidates for such immunointervention [4–6]. 

The preclinical period is marked by the presence of autoantibodies against beta cell antigens, including insulin, glutamic acid decarboxylase-65 (GAD65), insulinoma-associated tyrosine phosphatase (IA-2), and zinc transporter 8 (ZnT8). The presence of these autoantibodies in the serum is highly predictive of T1D development [7–9]. However, the presence of autoantibodies alone is not sufficient to induce the destruction of beta cells [10–13]. 

The preclinical disease stage is characterised by the generation of activated, self-reactive lymphocytes that infiltrate the pancreas and selectively destroy the insulin-producing beta cells present in the islets [14]. In addition, other cellular immune mechanisms including immunoregulation and antigen presentation and processing are involved in T1D pathogenesis. Other studies have revealed the importance of the failure of regulatory mechanisms, which mainly include regulatory T cells, which suppress proliferation and cytokine production by both CD4^+^ and CD8^+^ T cells *in vitro* in a cell contact-dependent manner, and the secretion of anti-inflammatory cytokines (e.g., interleukin- (IL-) 10 and transforming growth factor- (TGF-) beta) [15]. Taken together, T1D pathogenesis is very complex, and all aspects of this disease are not fully understood. Although autoantibody detection is very helpful in the study of this disease, this method is not sufficient for the identification of a prediabetic person. 

Autoreactive T lymphocytes are present in peripheral blood at extremely low frequencies, and methods for their detection are still used for scientific, rather than clinical, purposes [10, 13]. 

The last decade has ushered in a boom of “array techniques” that enable complex analyses of gene expression or protein production. These methods have also been used in T1D research to improve the prediction of T1D and increase the general knowledge of T1D pathogenesis [16, 17]. 

In this paper, we report the identification of cell processes that may be important for the progression of prediabetes to diabetes. We isolated peripheral blood mononuclear cells (PBMCs) and subsequently stimulated these cells with a mixture of “T1D-associated” autoantigens. We compared the expression profiles of stimulated PBMCs and PBMCs that were cultivated for the same period in the absence of autoantigens to determine the effect of autoantigens on gene expression. We describe, at the level of gene expression, the differences in the immune responses among the tested groups that are predicted to be important in T1D pathogenesis. Genes involved in these cascades, or in the activation of these cascades, may serve as promising potential prediabetes biomarkers. In our analyses, we primarily concentrated on functional pathways and attempted to reveal differences in gene expression among the multitude of signalling pathways within which these genes operate.

## 2. Materials and Methods

### 2.1. Study Subjects and Ethics

The study population is described in Table 1. Sera from all relatives were examined by radio-immune assay as a part of the national T1D prediction programme (RIA; Solupharm, Brno, Czech Republic) for the presence of autoantibodies against the islet antigens GAD65, IA-2, and insulin. The sample was considered positive if there was >1 IU/mL for GAD65 (GADA) and IA-2 (IA-2A) (>99th pct.). For insulin autoantibodies (IAAs), the cut-off was 0.4 U/mL. Autoantibody examination was successfully evaluated by the DASP 2010 (Diabetes Autoantibody Standardisation Programme of the Immunology of Diabetes Society). The type of autoantibody positivity in sera from patients and relatives is indicated in Supplementary Table 1s available online at http://dx.doi.org/10.1155/2013/589451. Sera from healthy volunteers were autoantibody negative.

The sampling of patients with a recent T1D onset was performed after the metabolic stabilisation phase on the seventh day after diabetes diagnosis in the morning hours. Metabolic stabilisation is defined as the establishment of normoglycaemia and the normalisation of acid-base balance, biochemical parameters (such as ions and pH), and blood count parameters. Patients with severe diabetic ketoacidosis (pH ≤ 7.1) at the time of the disease diagnosis were excluded from the study. The ethical approval, as well as the informed consent form, obligatory for all participants of this study, was processed by the Ethical Committee of the University Hospital Motol with respect to common national and EU rules. The patient's informed consent included blood sampling, isolation and analysis of nucleic acids, and anonymous data processing.

### 2.2. Cell Isolation and Stimulation by “T1D-Associated” Autoantigens

Approximately 17 mL of peripheral blood was obtained from the test subjects. PBMCs were isolated from whole venous blood by Ficoll density gradient centrifugation (Amersham Biosciences, Uppsala, Sweden) and were used in all *in vitro *experiments. The freshly isolated PMBCs (4 × 10^6^ cells) were resuspended in 2 mL of RPMI-1640 Medium (Invitrogen, Carlsbad, CA, USA) supplemented with 20% foetal calf serum (FCS-F7524, Sigma-Aldrich, St. Louis, USA) and 10 *μ*L/mL of Sigma solution, which contains 200 *μ*M L-glutamine, 100 U penicillin, and 100 *μ*g/mL streptomycin (G1146, Sigma-Aldrich, St. Louis, USA), and were cultured for 72 hours in the absence or presence of a mixture of the following autoantigen peptides (ProImmune, Oxford, UK): GAD65 amino acids (a.a.) 247–279 (NMYAMMIARFKMFPEVKEKGMAALPRLIAFTSEE-OH), molecular weight 3,823.7; a.a. 509–528 (IPPSLRTLEDNEERMSRLSK-OH), molecular weight 2,371.7; a.a. 524–543 (SRLSKVAPVIKARMMEYGTT-OH), molecular weight 2,238.7; IA-2 a.a. 853–872 (SFYLK (Nleu) VQTQETRTLTQFHF), molecular weight 2,489; and a.a. 9–23 of *β* proinsulin (SHLVEALYLVCGERG), molecular weight 1,645 at a concentration of 2 *μ*g/mL per 2 ∗ 10^6^ PBMCs for all autoantigen peptides. Length and amount of antigen exposure were optimising in laboratory (data not shown).

### 2.3. Nucleic Acid Isolation and Gene Expression Microarrays

Total RNA from cultured cells was extracted using TRIzol reagent and a RiboPure kit (Invitrogen, Carlsbad, CA, USA), dissolved in 60 *μ*L nuclease-free water and stored at −80°C. RNA concentration was measured using a spectrophotometer (Helios *γ*, Thermo Fisher Scientific, Waltham, MA, USA), and RNA integrity was assessed using an Agilent 2100 bioanalyser (Agilent, Palo Alto, CA, USA). To obtain a sufficient amount of RNA for the microarray assays, total RNA was amplified (aRNA) using the Amino Allyl MessageAmp II aRNA Amplification Kit (Applied Biosystems/Ambion, Foster City, CA, USA). The amplification procedure included the incorporation of 5-(3-aminoallyl)-UTP (aaUTP) into the aRNA during *in vitro *transcription to enable coupling of the RNA to N-hydroxysuccinimidyl ester-reactive Cy dyes. Twenty-five micrograms of aRNA was labelled with Cy3 or Cy5 dye. The Cy3 and Cy5 dyes were used to label RNA derived from nonstimulated and autoantigen-stimulated cells, respectively. From 3 to 6 *μ*g of labelled aRNA was hybridised to a chip (two colour experimental settings), according to the protocol of the manufacturer. Samples were then processed using a high-density human whole genome HOA gene array (Phalanx Biotech, Palo Alto, CA, USA) that contains 32,050 probes with 30,968 human genome targets and 1,082 experimental control probes. The slides were scanned using InnoScan 700 (Innopsys, Carbonne, France) at 5 *μ*m resolution. Artefacts were masked, and raw data were extracted using Mapix (Innopsys, Carbonne, France).

### 2.4. Gene Expression Microarray Data Analysis and Statistics

Microarray data processing and statistical analysis of differential gene expression was performed using the limma package in the R statistical environment (http://bioinf.wehi.edu.au/limma/), and a pathway analysis was performed with MetaCore (GeneGo, Inc., St. Joseph, MI, USA; http://www.genego.com/). Two-colour microarray data processing was performed as recommended by the array manufacturer. For each chip, raw intensity data were corrected for background, normalised by intra-array loess normalisation and subjected to subsequent interarray quantile normalisation. Differential gene expression was tested using the Bayesian moderated *t*-test in the limma package.

We examined differences in gene expression and affected cellular pathways between all combinations of the three groups: normal controls, diabetic patients, and their relatives, who were divided according to their autoantibody statuses. We compared basal gene expression with gene expression following stimulation with the diabetogenic autoantigens. The top table genes according to limma analysis (*P* value ≤ 0.05) were analysed by MetaCore to examine the functional relationships between the top genes (those genes with the most significant *P* values). We concentrated on identifying differences between tested pairs of study groups.

MetaCore is a proprietary, manually created database that analyses human protein-protein, protein-DNA, and protein-compound interactions, metabolic and signalling pathways, and the effects of bioactive molecules. This software generates interactive networks between user inputs and proteins and/or genes stored in the database. The software enables a user to analyse the distribution of canonical pathways, networks, GeneGo, and Gene Ontology processes, as well as the relevance of disease biomarkers in the tested samples. Canonical pathway maps represent a set of approximately 2,000 signalling and metabolic maps, comprehensively covering human biology. The content of approximately 110 cellular and molecular processes has been defined and annotated as GeneGo processes, and each process represents a preset network of interactions characteristic to the process. In this database, there are also over 500 human diseases with gene content annotated by GeneGo and organised in disease-specific folders, which are further organised into a hierarchical tree (http://www.genego.com/). We were interested in the general enrichment analysis and in the involvement of selected genes in immune processes, for which the data were filtered in the MetaCore Biomarker Assessment Workflow. 

### 2.5. qRT-PCR

qRT-PCR was used to verify microarray data. Differences in the expression levels of CD4, signal transducer and activator of transcription 3 (STAT3), and TGF-beta 1 between RNA samples from PMBCs collected from an independent cohort of T1D and the controls were assessed. Specifically, expression was analysed in a cohort of 14 newly diagnosed patients with T1D (7M/7F, mean age 8,6 years, median 9,1, range 1,7–17,2 years) and 12 control volunteers (5 M/7F, mean age 10,7 years, median 11,2, range 2,1–18,7 years) using TaqMan Gene Expression assays (Lifetechnologies, Carlsbad, CA, USA). Total RNA was extracted using TRIzol reagent, according to the manufacturer's recommendations (Lifetechnologies, Carlsbad, CA, USA). cDNA was synthesised according to recommendations by Lifetechnologies using the High Capacity RNA-to-cDNA Master Mix (Lifetechnologies, Carlsbad, CA, USA). Experiments were analysed using a LightCycler 480 Real-Time PCR System (Roche, Basel, Switzerland). A comparative ΔΔ cycle threshold (Ct) was used for quantification of relative mRNA levels. The expression of CD4 using commercially available primers (cat. no. Hs01058407_m1), STAT3 (cat. no. Hs00427259_m1), and TGF-beta (cat. no. Hs00171257_m1) was normalised to beta-glucuronidase (GUSB, cat. no. Hs99999908_m1). 

Data from the qRT-PCR were analysed using the R programme. An unpaired, two-tailed Student's *t*-test was used for statistical analysis. Differences with a *P* value ≤ 0.05 were considered significant.

## 3. Results

### 3.1. Expression of Single Genes

**Figure 1:**
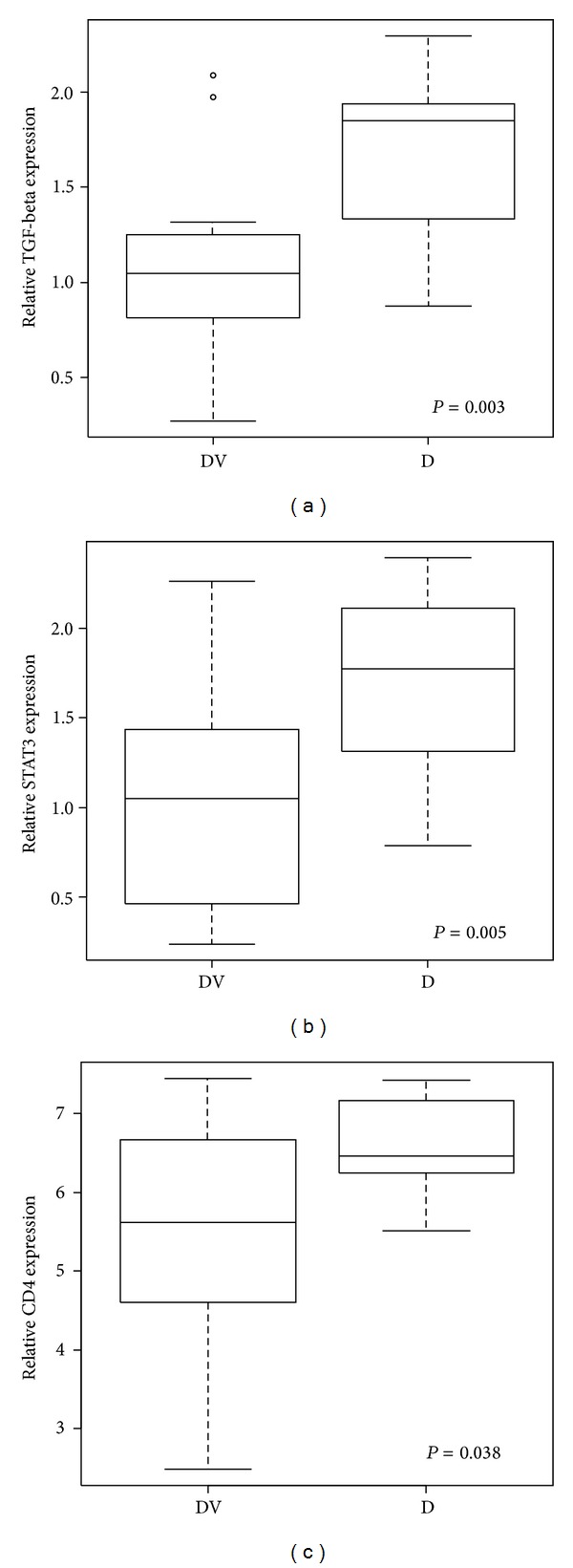
Verification of gene microarray data. Relative expression of TGF beta1, STAT3, and CD4 by qRT-PCR (data were obtained from an independent cohort of 14 newly diagnosed patients with T1D and 12 healthy volunteers).

Table 2 summarises the number of genes identified as having different expression levels when the various test groups were subjected to pair group comparisons. In the comparison of patients with T1D versus controls, statistically significant differences were present in the expression of 1,318 genes. The 20 genes that demonstrated the greatest changes in gene expression (up- or downregulated) are listed in Supplementary Table 2s. Interestingly, one of the most significantly upregulated genes in patients with T1D was CD4, a critical Lck-binding coreceptor required for the efficient activation of CD4^+^ T cells [18]. Using qRT-PCR, the differential expression of CD4 was confirmed on a separate cohort of newly diagnosed patients with T1D and healthy controls (Figure 1). In addition, TGF-beta and STAT3, representatives of Th17 cell differentiation signalling (which scored as the second most significantly changed immune-related pathway in T1D patients compared to healthy controls), were also confirmed to be significantly (*P* < 0.05) upregulated (Figure 1). 

Interestingly, the highest number of differentially expressed genes (2,222; *P* value ≤ 0.05) was found between relatives of TD1 patients and patients. A list of the top 20 up- and downregulated genes identified can be found in Supplementary Table 2s. Moreover, the relatives had significant alterations in the expression of 1,347 genes compared to controls (Supplementary Table 2s). However, we were unable to find any additional significant differences in gene expression when the relatives were divided according to autoantibody status in the DRLP (autoantibody/ies positive) and DRLN (autoantibody/ies negative) groups. 

**Figure 2:**
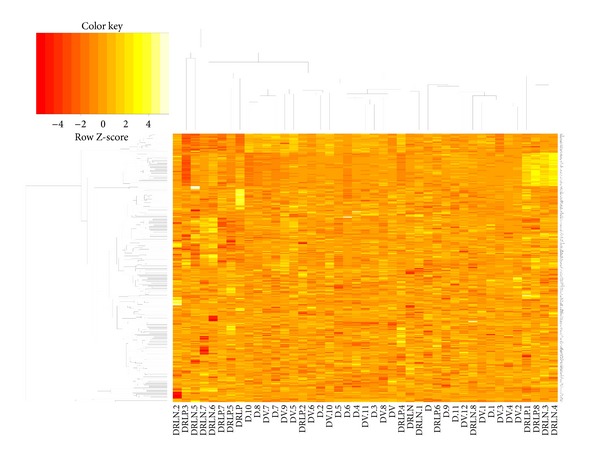
Genes differentially activated in each group (cluster formation). The enhanced gene expression heatmap was constructed using probe signal intensities that had a log fold change that was greater than +1 or less than −1. Genes that were significantly altered in the relatives group clustered into specific gene families.

An enhanced gene expression heatmap was constructed using probe signal intensities that had a log fold change that was greater than +1 or less than −1 (Figure 2).

### 3.2. Functional Genomics

The top ten canonical pathways that changed most significantly in the pair-wise comparisons are listed in Table 3(a) (summary), and Table 3(b) shows the complete list of significant immune response pathways identified for each pair-wise comparison. 

**Figure 3:**
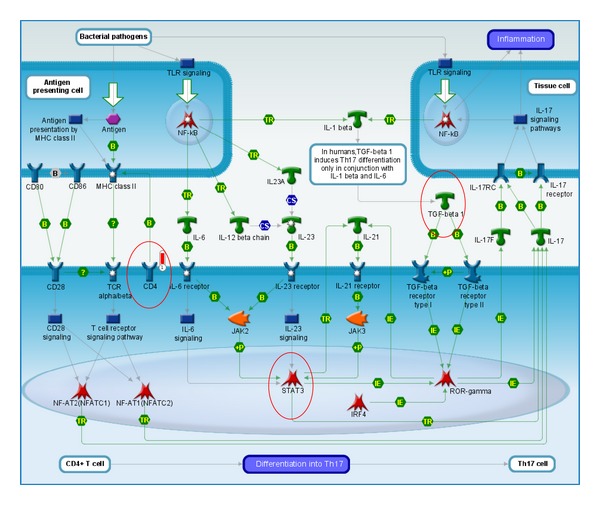
Immune response and Th17 cell differentiation. Differences in Th17 polarisation were observed when controls were compared with T1D patients using microarray data. Genes of interest were analysed by qRT-PCR and were found to be upregulated in T1D patients. STAT3 and TGF-beta were chosen as representatives of Th17 cell differentiation. Microarray data demonstrated that CD4 was one of the most significantly upregulated molecules in T1D patients.

The greatest number of differences for pathways that were altered, specifically 69 pathways, was observed when relatives were compared to controls. Of these pathways, 15% belonged to “Immune response pathways.” However, the highest percentage (24%) of significant differences in immune response-related pathways was observed when patients with T1D were compared with healthy controls (11 out of 46 pathways), with “Antigen presentation by MHCII” as the highest scoring pathway. An important variable appeared to be Th17 lymphocyte activation, as we observed a difference in “Th17 cell differentiation” among the groups. Specifically, differences in Th17 polarisation were observed when relatives were compared with patients. The Th17 cell differentiation pathway is shown in Figure 3. Additionally, by comparing patients with T1D with the control group, we observed the distinct activation of important immune pathways involved in specific immune responses, such as Th1/Th2 polarisation, the formation of immunological synapses, and signalling via the T cell receptor (Table 3(b)). 

Immunologic responsiveness in relatives was similar to the responsiveness observed in patients with T1D. However, only 7% of the differentially activated pathways could be classified as “immune response-related” (i.e., 4 pathways out of 54 differentially activated pathways). Within these pathways, cell cascades related to Th17 polarisation and the action of the immunoregulatory cytokine TGF-beta were also identified. 

## 4. Discussion

Upon activation and expansion, naive CD4^+^ T cells develop into different Th cell subsets that exhibit different cytokine profiles and effector functions to protect the body against different types of pathogens. Until recently, T cells were divided into Th1 and Th2 cells, depending on the cytokines they produced (e.g., IFN-gamma and TNF-beta versus IL-4, -5, and -13, resp.). 

A third subset of IL-17-producing effector Th cells called Th17 cells has recently been discovered. The participation of TGF-beta in Th17 cell differentiation places the Th17 lineage in close relationship with CD4^+^CD25^+^Foxp3^+^ regulatory T cells (Tregs) [19].

T1D is an autoimmune disease that results from the selective destruction of pancreatic beta-cells by T cells, and the development of this disease is most likely due to the interaction between environmental and genetic factors. CD4^+^ T cells are largely implicated in the pathogenesis of this disease, and T1D is believed to be a predominantly Th1-driven disease. Moreover, increased IL-17 expression has been detected in the sera and target tissues of patients with various autoimmune diseases, and in animal models, IL-23, a Th17 stabilisation factor, is involved in the development of autoimmune diabetes. The differentiation of Th17 cells is initiated by TGF-beta, IL-6, and IL-21, which activate STAT3 and induce the expression of transcription factors, including retinoic acid related orphan receptor (RORgamma t). In humans, Th17 activity seems to cause multiorgan inflammation, contributing to the manifestation of rheumatoid arthritis, inflammatory bowel disease, and celiac disease [20]. 

In this unique study on gene expression and functional analysis, we demonstrated that the “Th17 differentiation,” “IL-22 signalling,” and “Development of TGF-beta receptor signalling” pathways were among the most significantly different pathways identified when patients with T1D were compared with healthy controls. A difference in “Th17 signalling” pathway activation was also observed when we compared T1D patients with relatives. Consistent with these data, we previously reported that a bias in IL-10 and TGF-beta production at the protein level is typical of the prediabetes phase [21, 22]. 

Using a murine model of the disease, two groups previously reported that the transfer of islet-specific Th17 cells induced diabetes, although this effect was apparent only after the cells had converted to IFN-producing cells [23, 24]. Although TGF-beta and IL-21 can cause naive CD4^+^ cells to differentiate into Th17 cells that secrete IL-17 in humans, it has been demonstrated that central memory CD4^+^ cells can be driven to secrete IL-17 by a combination of IL-1 and IL-6 [25–28]. Bradshaw and colleagues studied monocytes directly isolated from the blood of patients with T1D and found that the cells spontaneously secreted the proinflammatory cytokines IL-1 beta and IL-6, which are known to induce and expand Th17 cells. Moreover, these *in vivo* activated monocytes induced more IL-17-secreting cells from memory T cells compared to monocytes from healthy control subjects. The induction of IL-17-secreting T cells by monocytes from patients with T1D was reduced *in vitro* with a combination of an IL-6-blocking Ab and an IL-1R antagonist. In this study, the authors also reported a significant increase in the frequency of IL-17-secreting cells in lymphocytes from long-term patients with T1D compared to healthy controls. These data suggest that the innate immune system in T1D patients may drive the adaptive immune system by expanding the Th17 population of effector T cells [29]. Consistent with the results of this report, our data also suggest that a “Th17 bias” may be present many years after disease onset and indicate the existence of a certain “autoreactive potential” of the immune system.

IL-9 is a T cell-derived cytokine that was initially characterised as a Th2 cytokine. The secretion of IL-9 was recently attributed to a novel CD4^+^ T cell subset termed Th9 cells in mice. However, IL-9 can also be secreted by mouse Th17 cells and may mediate aspects of the proinflammatory activities of Th17 cells [30]. Beriou and colleagues reported that IL-9 is secreted by human naive CD4^+^ T cells in response to differentiation under Th9- (i.e., TGF-beta and IL-4) or Th17- (i.e., TGF-beta and IL-6) polarising conditions. Yet, these differentiated naive cells did not coexpress IL-9 and IL-17 unless the cells were repeatedly stimulated under Th17 differentiation-inducing conditions. These authors demonstrated that patients with autoimmune diabetes exhibit higher frequencies of memory CD4^+^ T cells and that activation of these cells in the presence of TGF-beta induces a memory CD4^+^ T cell response that is dominated by IL-9 and IL-17, accompanied by a loss of Th1 and Th2 cytokines. These data demonstrate that the presence of IL-9^+^ IL-17^+^ CD4^+^ T cells induced by IL-1 beta may play a role in human autoimmune disease [30]. 

Not surprisingly, the highest scoring pathway in the comparison of patients with T1D versus their healthy counterparts was “Antigen presentation by MHCII”; indeed, it is well known that genes encoding HLA class II molecules are the most important “T1D-associated genes” [10]. It is also not surprising that other pathways related to crucial processes of the specific immune response, such as the “T cell receptor signalling pathway,” demonstrated differences in activation in patients with T1D. Similarly, significant differences in Rho family GTPase signalling, namely, the Rac3 and Cdc42 pathways, which regulate cytoskeletal organisation and membrane trafficking and have been proposed to be linked to diabetes [31], were among the top ten pathways scored. 

Glucose-stimulated insulin secretion from islet beta-cells involves secretory granule transport, a highly coordinated process that involves changes in cytoskeletal architecture with the help of G proteins and their respective effector molecules. Small G proteins include Cdc42, Rac1, and ARF-6, with corresponding regulatory factors including GDP/GTP-exchange factors and GDP-dissociation inhibitors. In addition to their positive modulatory roles, certain small G proteins also contribute to the metabolic dysfunction and the demise of islet beta-cells that has been observed in *in vitro* and *in vivo* models of impaired insulin secretion and diabetes [32]. 

The bone morphogenic protein (BMP) signalling pathway also appeared on the list of differentially activated pathways when patients were compared with controls and also with relatives. It is well known that diabetic nephropathy is a leading cause of end-stage renal disease. Additionally, the TGF-beta-BMP pathway has been implicated in the pathogenesis of diabetic nephropathy. The BMP2, BMP4, and BMP7 genes are located near linkage peaks for renal dysfunction, and it was hypothesised that genetic polymorphisms in these biological and positional candidate genes may constitute a risk factor for diabetic kidney disease; however, common BMP gene polymorphisms do not strongly influence genetic susceptibility to diabetic nephropathy in white individuals with T1D [33]. None of the tested patients had diabetic nephropathy at the time of sampling, but there may be a correlation between these symptoms and a higher risk of the development of chronic diabetic complications. Recently, it has also been suggested that TGF-beta/BMP-6 signalling in diabetic patients contributes to enhanced cell differentiation of circulating smooth muscle progenitor cells [34]. 

There have been only a limited number of T1D gene expression studies. One example is the report by Kaizer and colleagues [16] who analysed the gene expression of PBMCs derived from paediatric patients with T1D and T2D. The authors found that T1D and T2D likely share a downstream common pathway for beta-cell dysfunction that includes secretion of IL-1 beta and prostaglandins by immune effector cells, although the authors did not test the effect of autoantigen stimulation. In the Czech Republic, T2D is rare in children; therefore, we did not compare our data with data obtained from T2D patients, who typically belong to a more aged population. Reynier and colleagues tested first-degree relatives of T1D patients, but these authors also did not incorporate autoantigen exposure into their experiments, similar to Kaizer et al. [16]. Thus, our study appears quite unique in the sense that it compares the effects of autoantigen stimulation on cell processes in PMBCs in the normal and autoimmune diabetes states.

One potential drawback to our study is the limited number of samples tested. However, we believe that approximately ten subjects per group are sufficient to reveal genes with statistically significant alterations in their gene expression levels when high-density microarray chips are used. In this context and in many other aspects, the results of this study parallel our previous work [35] and the studies of other research teams in which microarray analyses obtained from a limited number of subjects provided highly relevant and statistically significant data [16, 36–38]. Moreover, while our control group was not ideally age-matched to our other study groups, this variable produces negligible effects on our statistical analyses (data not shown) according to our comprehensive statistical analysis described elsewhere [35]. As an example, our assessment of the impact of age and sex on the expression of CD4 was statistically insignificant. 

In conclusion, we can summarise that important differences were observed when the activation of cell processes following artificial exposure to diabetes-related autoantigens was compared among T1D patients, their first-degree relatives, and healthy controls. Important immune response-related pathways were involved, with a high degree of variability observed for these pathways when either patients with T1D or their relatives were compared with healthy controls. These important immune response-related processes largely included the induction of Th17 and Th22 responses, as well as cytoskeletal rearrangements, MHCII presentation, and the upregulation of CD4, TGF-beta, and STAT3. These findings potentially suggest that these processes could be utilised as predictive markers for the development of T1D or as molecular targets for the repression of specific immunocompetent cell populations for the treatment of diabetes. 

## Supplementary Material

Detailed description of autoantibodies status of study cohort could be found in Table 1s. Greatest changes in a gene expression of 20 up- or downregulated genes among our groups are listed in a Table 2s.

## Figures and Tables

**Table 1:** Study population.

Study group	No. of individuals	Age (years) median, range	Age (years)	Sex (F/M)
T1D recent onset	12	12; 3–41	12 12 3 17 12 8 7 41 19 15 7 7	M M F M F F M M M F F F

First-degree relatives autoantibodies negative	9	19; 5–52	5 7 32 52 43 19 22 16 8	F F F M F F M M F

First-degree relatives autoantibodies positive	9	7; 3–21	7 13 10 7 3 7 7 21 7	F F M F M F F F F

Controls	13	27; 14–42	14 36 22 22 21 21 27 31 27 42 27 32 24	M F F F F M F M M M F F M

**Table 2:** The number of identified genes with different expression levels when the various test groups were subjected to pair group comparisons.

Comparison	Total no. of sign. differentially activated genes	No. of sign. upregulated genes	No. of sign. downregulated genes
DRL versus D	2222	1513	709
DV versus D	1318	896	422
DRL versus DV	1347	955	392

D: T1D patients; DRL: first-degree relatives of T1D patients; DV: controls (healthy volunteers).

**Table:** (a)

T1D (D) patients versus healthy controls (DV)	Relatives of T1D patients (DRL) versus healthy controls (DV)	T1D (D) patients versus relatives of T1D patients (DRL)
**(1) Immune response_Antigen presentation by MHCII** (2) G protein signalling_Rac3 regulation pathway (3) Neurophysiological process_Olfactory transduction (4) Transcription_CREM signalling in testis (5) Dichloroethylene metabolism (6) Delta508-CFTR traffic/sorting endosome formation in CF **(7) Immune response_Th17 cell differentiation** (8) G-protein signalling_Regulation of CDC42 activity **(9) Immune response_IL-22 signalling pathway** (10) Development_BMP signalling	**(1) Immune response_MIF-JAB1 signalling** (2) Cytoskeleton remodeling_Fibronectin bindings integrins in cell motility (3) Translation_(L)-selenoaminoacids incorporation in proteins during translation (4) Regulation of lipid metabolism_Insulin regulation of glycogen metabolism (5) Glutathione metabolism (6) Development_Ligand-dependent activation of the ESR1/AP1 pathway (7) G protein signalling_Rac3 regulation pathway (8) Protein folding_Membrane trafficking and signal transduction of G-alpha (9) Neurophysiological process_Olfactory transduction (10) Dichloroethylene metabolism	(1) Cytoskeleton remodeling_CDC42 in cellular processes (2) Development_BMP signalling (3) Neurophysiological process_EphB receptors in dendritic spine morphogenesis and synaptogenesis (4) Development_Hedgehog signalling (5) Neolacto-series GSL Metabolism p.2 and p.3 (6) Neurophysiological process_Olfactory transduction (7) Atherosclerosis_Role of ZNF202 in regulation of expression of genes involved in Atherosclerosis (8) Dichloroethylene metabolism (9) Cytoskeleton remodeling_Neurofilaments (10) Triacylglycerol metabolism p.2

**Table:** (b)

T1D (D) patients versus healthy controls (DV)	Relatives of T1D patients (DRL) versus healthy controls (DV)	T1D (D) patients versus relatives of T1D patients (DRL)
1: Antigen presentation by MHCII 7: Th17 cell differentiation 9: IL-22 signalling pathway 20: TCR and CD28 costimulation in activation of NF-kB 23: Th1 and Th2 cell differentiation 25: HTR2A induced activation of cPLA2 28: IL-13 signalling via JAK-STAT 32: Lectin induced complement pathway 33: Development of TGF-beta receptor signalling 35: T cell receptor signalling pathway 41: Immunological synapse formation	1: MIF-JAB1 signalling 27: CXCR4 signalling via second messenger 28: CXCR4 signalling pathway 32: Regulation of T cell function by CTLA-4 36: IL-7 signalling in T lymphocytes 43: IL-7 signalling in B lymphocytes 53: T cell receptor signalling pathway 55: CD28 signalling 56: Role of DAP12 receptor in NK cells 59: Immunological synapse formation	24: Cytokine production by Th17 cells 31: TGF-beta receptor signalling 36: Th17 signalling pathway 40: Gastrin in inflammatory response

D: T1D patients; DRL: first-degree relatives of T1D patients; DV: controls (healthy volunteers).

## Legend for the Tables and Figures

D: T1D patients
DRL: First-degree relatives of T1D patients
DRLN: Relatives of T1D patients who are autoantibody(ies) negative
DRLP: Relatives of T1D patients who are autoantibody(ies) positive
DV: Controls (healthy volunteers)
FC: Fold change
GADA: Antiglutamic acid decarboxylase (GAD65) autoantibodies
IA-2A: Antityrosin phosphatase (IA-2) autoantibodies
IAA: Insulin autoantibodies.
