# Supplementary material for: The Effect of Diabetes-Associated Autoantigens on Cell Processes in Human PBMCs and Their Relevance to Autoimmune Diabetes Development
Source: J Diabetes Res. 2013 Jun 12;2013:589451. doi: 10.1155/2013/589451 (PMC3694381; doi:10.1155/2013/589451)
Supplement: Supplementary file 1 — Detailed description of autoantibodies status of study cohort could be found in Table 1s. Greatest changes in a gene expression of 20 up- or downregulated genes among our groups are listed in a Table 2s. [file 589451.f1.pdf]

Table 1s

Further characterisation of T1D patients and relatives who were autoantibody positive

**T1D patients**		
**Sex**	**Age (years)**	**Autoantibody status**
**F**	**3**	**IAA**
**F**	**5**	**IAA, IA-2A**
**F**	**5**	**IAA, IA-2A**
**F**	**14**	**IA-2A, GADA**
**F**	**13**	**IA-2A, GADA**
**F**	**15**	**GADA**
**M**	**4**	**IA-2A**
**M**	**7**	**IA-2A**
**M**	**13**	**IA-2A, GADA**
**M**	**14**	**IA-2A, GADA**
**M**	**14**	**IA-2A, GADA**
**M**	**41**	**GADA**
**Relatives**		
**F**	**3**	**IAA**
**F**	**21**	**IA-2A, GADA**
**M**	**3**	**IAA, IA-2A**
**M**	**4**	**IA-2A, GADA**
**M**	**5**	**IA-2A**
**M**	**5**	**IA-2A, GADA**
**M**	**7**	**IA-2A, GADA**
**M**	**8**	**IA-2A, GADA**
**M**	**10**	**IA-2A, GADA**

Table 2s

Top twenty up- or downregulated genes for each comparison

UPREGULATED **First group is always upregulated in comparison do the other one.**			
**Entrez_Gene_Description**	**Entrez_Gene_Name**	**Fold_change**	**P_Value**
**DV vs D**			
renin	*REN*	2.177	0.002
melanoma antigen, family C, 1	*MAGEC1*	2.001	0.002
glycoprotein hormone beta 5	*GPHB5*	1.918	0.000
relaxin 3 receptor 1	*RLN3R1*	1.866	0.000
homeo box (H6 family) 3	*HMX3*	1.832	0.001
tripartite motif-containing 67	*TRIM67*	1.817	0.000
zinc finger protein 560	*ZNF560*	1.814	0.000
carboxypeptidase E	*CPE*	1.813	0.001
DKFZP434A062 protein	*DKFZP434A062*	1.809	0.001
late cornified envelope 2B	*LCE2B*	1.798	0.000
cholinergic receptor, nicotinic, gamma polypeptide	*CHRNG*	1.789	0.001
phosphatidylinositol-specific phospholipase C, X domain containing 2	*PLCXD2*	1.722	0.002
dystrophin related protein 2	*DRP2*	1.708	0.001
polycystic kidney disease 1-like 2	*PKD1L2*	1.702	0.001
ATP-binding cassette, sub-family A (ABC1), member 4	*ABCA4*	1.693	0.001
similar to developmental pluripotency associated 5; embryonal stem cell specific gene 1	*LOC340168*	1.689	0.001
similar to precursor peptide	*LOC340204*	1.685	0.001
growth differentiation factor 8	*GDF8*	1.677	0.000
similar to RIKEN cDNA 2410004A20	*LOC154288*	1.666	0.001
similar to Pregnancy specific beta-1-glycoprotein 4, isoform 1	*LOC440534*	1.635	0.001
**DRLvs DV**			
ATPase, (Na+)/K+ transporting, beta 4 polypeptide	*ATP1B4*	1.935	0.001

scavenger receptor cysteine rich domain containing, group B (4 domains)	*SRCRB4D*	1.893	0.000
dynein, axonemal, heavy polypeptide 8	*DNAH8*	1.732	0.001
programmed cell death 2	*PDCD2*	1.681	0.003
aldo-keto reductase family 1, member C-like 1	*AKR1CL1*	1.664	0.001
integrin, alpha 10	*ITGA10*	1.638	0.000
chromosome 10 open reading frame 92	*C10orf92*	1.622	0.002
chromosome 8 open reading frame 15	*C8orf15*	1.583	0.004
C1q and tumor necrosis factor related protein 3	*C1QTNF3*	1.549	0.003
F-box and leucine-rich repeat protein 14	*FBXL14*	1.519	0.002
CD5 antigen-like (scavenger receptor cysteine rich family)	*CD5L*	1.502	0.002
tripartite motif-containing 55	*TRIM55*	1.473	0.004
H2A histone family, member J	*H2AFJ*	1.459	0.002
parathyroid hormone	*PTH*	1.456	0.003
chromosome 21 open reading frame 13	*C21orf13*	1.426	0.005
similar to sphingomyelin phosphodiesterase 3, neutral membrane	*LOC392275*	1.423	0.003
serologically defined colon cancer antigen 33	*SDCCAG33*	1.387	0.002
minor histocompatibility antigen HA-1	*HA-1*	1.380	0.001
dual specificity phosphatase 8	*DUSP8*	1.365	0.001
proprotein convertase subtilisin/kexin type 5	*PCSK5*	1.361	0.003
**DRLvs D**			
kelch domain containing 4	*KLHDC4*	1.586	0.000
growth differentiation factor 8	*GDF8*	1.673	0.000
similar to precursor peptide	*LOC340204*	1.815	0.000
glial fibrillary acidic protein	*GFAP*	1.911	0.000
cholinergic receptor, nicotinic, gamma polypeptide	*CHRNG*	1.921	0.000
hepatocyte nuclear factor 4, alpha	*HNF4A*	1.499	0.000
colipase, pancreatic	*CLPS*	1.799	0.000
chromosome 11 open reading frame 33	*C11orf33*	1.565	0.000
crystallin, gamma D	*CRYGD*	1.635	0.000
MEGF11 protein	*MEGF11*	1.519	0.000
FLJ16237 protein	*FLJ16237*	1.656	0.000

serine (or cysteine) proteinase inhibitor, clade B (ovalbumin), member 2	*SERPINB2*	2.368	0.000
a disintegrin-like and metalloprotease (reprolysin type) with thrombospondin type 1 motif, 19	*ADAMTS19*	1.690	0.001
gap junction protein, alpha 10, 59kDa	*GJA10*	1.682	0.001
folate receptor 2 (fetal)	*FOLR2*	2.623	0.001
relaxin 3 receptor 1	*RLN3R1*	1.708	0.001
transient receptor potential cation channel, subfamily C, member 6	*TRPC6*	1.484	0.001
GCIP-interacting protein p29	*P29*	1.572	0.001
NADH:ubiquinone oxidoreductase MLRQ subunit homolog	*LOC56901*	1.384	0.001
chemokine (C-X-C motif) ligand 1 (melanoma growth stimulating activity, alpha)	*CXCL1*	1.816	0.001

DOWNREGULATED **First group is always downregulated in comparison do the other one.**			
**Entrez_Gene_Description**	**Entrez_Gene_Name**	**Fold_ change**	**P_Value**
**DV vs D**			
solute carrier family 7 (cationic amino acid transporter, y+ system), member 3	*SLC7A3*	0.464	0.002
transient receptor potential cation channel, subfamily V, member 3	*TRPV3*	0.472	0.005
ring finger protein 144	*RNF144*	0.484	0.001
collagen, type XVI, alpha 1	*COL16A1*	0.543	0.004
CD4 antigen (p55)	*CD4*	0.557	0.002
LOC441491	*LOC441491*	0.562	0.006
glomulin, FKBP associated protein	*GLMN*	0.586	0.006
tumor susceptibility gene 101	*TSG101*	0.590	0.005
chromosome 6 open reading frame 122	*C6orf122*	0.596	0.006
kinesin family member 27	*KIF27*	0.599	0.002
ankyrin repeat and KH domain containing 1	*ANKHD1*	0.600	0.003
keratin associated protein 4-5	*KRTAP4-5*	0.607	0.003
myotubularin related protein 7	*MTMR7*	0.648	0.003
zinc finger protein 266	*ZNF266*	0.653	0.005
FLJ42258 protein	*FLJ42258*	0.654	0.001

sarcoma antigen 1	*SAGE1*	0.658	0.002
chromosome 20 open reading frame 23	*C20orf23*	0.665	0.006
cytochrome P450, family 2, subfamily R, polypeptide 1	*CYP2R1*	0.666	0.004
testis specific, 10 interacting protein	*TSGA10IP*	0.679	0.006
solute carrier family 24 (sodium/potassium/calcium exchanger), member 1	*SLC24A1*	0.680	0.006
**DRLvs DV**			
heparan sulfate (glucosamine) 3-O-sulfotransferase 2	*HS3ST2*	0.320	0.001
renin	*REN*	0.516	0.003
melanoma antigen, family C, 1	*MAGEC1*	0.522	0.002
KIAA1411	*KIAA1411*	0.528	0.004
deafness, autosomal dominant 5	*DFNA5*	0.530	0.001
RAB17, member RAS oncogene family	*RAB17*	0.532	0.000
tryptophan hydroxylase 2	*TPH2*	0.546	0.001
KIAA1889 protein	*KIAA1889*	0.569	0.002
chromosome 12 open reading frame 4	*C12orf4*	0.581	0.001
D21S2091E	*D21S2091E*	0.592	0.000
glutathione peroxidase 2 (gastrointestinal)	*GPX2*	0.619	0.004
tripartite motif-containing 67	*TRIM67*	0.628	0.001
myomesin (M-protein) 2, 165kDa	*MYOM2*	0.631	0.001
Jak and microtubule interacting protein 2	*KIAA0555*	0.633	0.001
zinc finger protein 560	*ZNF560*	0.635	0.000
amiloride-sensitive cation channel 1, neuronal (degenerin)	*ACCN1*	0.637	0.003
a disintegrin-like and metalloprotease (reprolysin type) with thrombospondin type 1 motif, 1	*ADAMTS1*	0.646	0.003
olfactory receptor, family 11, subfamily A, member 1	*OR11A1*	0.652	0.002
KCNQ1 downstream neighbor	*KCNQ1DN*	0.656	0.002
regulator of G-protein signalling 13	*RGS13*	0.658	0.003
**DRLvs D**			
heparan sulfate (glucosamine) 3-O-sulfotransferase 2	*HS3ST2*	0.280	0.000
solute carrier family 7 (cationic amino acid transporter, y+ system), member 3	*SLC7A3*	0.432	0.000
HtrA serine peptidase 4	*HTRA4*	0.436	0.000
GM2 ganglioside activator	*GM2A*	0.447	0.000
leukocyte immunoglobulin-like receptor, subfamily A (with TM domain), member 2	*LILRA2*	0.501	0.001
apolipoprotein C-I	*APOC1*	0.503	0.002
deafness, autosomal dominant 5	*DFNA5*	0.509	0.000

phosphatidic acid phosphatase type 2B	*PPAP2B*	0.525	0.001
bone morphogenetic protein 6	*BMP6*	0.530	0.002
GM2 ganglioside activator	*GM2A*	0.536	0.000
RAB17, member RAS oncogene family	*RAB17*	0.550	0.000
pleckstrin and Sec7 domain containing 3	*PSD3*	0.560	0.002
coronin, actin binding protein, 1C	*CORO1C*	0.579	0.000
vesicle amine transport protein 1 homolog (T californica)	*VAT1*	0.579	0.000
homeo box D4	*HOXD4*	0.612	0.001
sema domain, immunoglobulin domain (Ig), short basic domain, secreted, (semaphorin) 3C	*SEMA3C*	0.613	0.001
keratin associated protein 4-5	*KRTAP4-5*	0.614	0.002
sialyltransferase 10 (alpha-2,3-sialyltransferase VI)	*SIAT10*	0.626	0.000
ferredoxin 1	*FDX1*	0.634	0.000
cytochrome P450, family 2, subfamily R, polypeptide 1	*CYP2R1*	0.641	0.001
